# A Case of Metastatic Head and Neck Squamous Cell Carcinoma with Multiple Treatments Including Stereotactic Ablative Body Radiotherapy Alone for Oligometastases to the Parotid Gland

**DOI:** 10.7759/cureus.438

**Published:** 2015-12-28

**Authors:** Jonathan Sharrett, Aaron Pederson

**Affiliations:** 1 Internal Medicine, Memorial Health University Medical Center; 2 Radiation Oncology, Memorial Health University Medical Center

**Keywords:** oligo-metastatic, head and neck cancers, stereotactic ablative radiotherapy (sabr), metastatic, chemoradiation, surgical resection, srs and sbrt treatment

## Abstract

Head and neck squamous cell carcinoma (HNSCC) has a reported average of around 15% metastases at presentation with chemotherapy being the mainstay of treatment for widely metastatic disease. However, in select patients with oligometastatic disease, local ablative therapy (commonly including surgery or radiotherapy) can be utilized with the possibility of improving survival, decreasing morbidity from the metastases, and obviating the need for systemic therapy with its possible side effects. However, most research has been of ablative therapy has been performed for pulmonary and hepatic oligometastatic lesions.

In this case, we present a patient who initially presented with a metastatic base of tongue malignancy with left axilla metastases who was treated palliatively with systemic therapy with an initial complete response. She subsequently progressed on maintenance therapy with a locoregional recurrence that was treated with definitive chemoradiation with complete response. There was a subsequent recurrence again in the left axilla that was treated as well with chemoradiation with complete response. Next, there was a recurrence in the right occipital lobe and left parotid gland with treatment with surgical resection, plus stereotactic radiotherapy boost and stereotactic ablative body radiotherapy (SABR), respectively. This all occurred over a 30-month time frame from initial therapy to her last treatment, with an additional 42 months at the present time with no recurrence.

This case highlights the future of care of oligometastatic disease of HNSCC with potential long-term survival in appropriately selected patients treated with stereotactic ablative body radiotherapy. Furthermore, this is one of the first reported cases in the literature of SABR for an oligometastatic lesion of the parotid gland, especially from squamous cell carcinoma (SCC) of the oral cavity, with no recurrence of disease over 40 months removed from treatment.

## Introduction

Head and neck squamous cell carcinoma (HNSCC) has a reported average metastatic potential on presentation of around 15% based on recent analysis [1]. Chemotherapy, with or without molecularly targeted therapy, has been the standard of care of HNSCC distant metastatic disease [2]. This is typically performed with palliative intent as survival is on average only 7-10 months and less than 10-15% are alive at five years, with the most significant increase in survival over the past 30 years due to the addition of cetuximab to platinum-based chemotherapy [2].  

Oligometastatic disease is defined as one to five metastatic lesions besides the primary tumor and likely represents an intermediate stage between local and widespread metastases [3]. Local ablative therapy has had an increasing role in the treatment of oligometastatic disease, with the hopes of improving survival and decreasing the need for toxic systemic therapy.

The most common site of metastatic disease in HNSCC is lung, which accounts for two-thirds of cases, with bone being the next most common, followed by liver, while skin, mediastinum, and bone marrow are other less uncommon sites [4-5]. Parotid metastasis is an uncommon location for HNSCC. The standard treatment for all parotid metastases has been surgical resection with a case series of adjuvant radiation therapy in select patients [6]. Evidence for radiation therapy alone for metastatic HNSCC to the parotid gland is lacking or for any metastatic lesion to the parotid.

We herein report a case of metastatic HNSCC initially treated with palliative systemic therapy achieving a complete response that subsequently recurred multiple times requiring different treatment modalities. The last recurrence was a parotid metastasis treated by means of stereotactic ablative body radiotherapy instead of surgical resection. The patient has maintained complete remission status now 40 months from his last treatment and 72 months from the initial diagnosis.

## Case presentation

Informed patient consent was obtained for the treatment of this patient. No identifying patient information is disclosed in this study.

A 64-year-old female was diagnosed with Stage IVC, T4aN2cM1 squamous cell carcinoma (SCC) of the right base of the tongue approximately six years ago in 2009 with metastases to the left axilla based on positive PET scan and biopsy. She began palliative systemic therapy and completed seven weekly cycles of carboplatin, paclitaxel, and cetuximab. She had a follow-up PET-CT scan around three months later in the spring of 2010 that was negative. She was continued on maintenance cetuximab every two weeks at that time. Three months later, she had a repeat follow-up PET-CT scan demonstrating uptake at the primary site at the base of tongue and in the bilateral neck. Biopsy of a palpable neck lymph node demonstrated SCC. There was no evidence of any distant metastatic disease detected by PET-CT scan.

She received definitive chemoradiation to the bilateral uninvolved neck to 60 Gy in 2 Gy daily fractions with a simultaneous boost to areas of gross disease to 66 Gy in 2.2 Gy daily fractions. She received concurrent chemotherapy with 5-Flurouracil and cisplatin with a complete response and no evidence of disease (NED) on repeat imaging. Around eight months after completion of therapy, with no interval clinical and radiographic evidence of disease, a follow-up PET-CT showed two PET-avid lymph nodes in the left axilla at levels I and II, with the largest being 1.5 cm and was biopsy-proven to be metastatic SCC. The patient opted for aggressive chemoradiation and was treated with concurrent Xeloda and IMRT external beam radiation with 50 Gy to the left axilla with an in-field boost to 66 Gy. Three months after treatment, there was NED on PET-CT.

**Figure 1:**
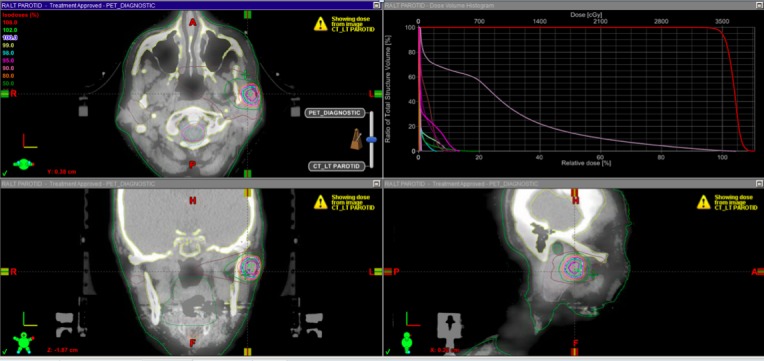
Stereotactic Ablative Body Radiotherapy Treatment Layout and Dose-Volume Histogram for Right Parotid Gland Oligometastatic Lesion

Another three months passed, taking us to December 2011, and, unfortunately, follow-up PET-CT showed concern for a left parotid gland metastasis. She additionally had an MRI of the brain that showed an enhancing lesion in the right occipital lobe. First, she underwent craniotomy with a complete resection of the tumor, which was confirmed to be SCC. She received a stereotactic radiosurgery (SRS) boost with 8 Gy x 3 fractions for a total of 24 Gy to the right occipital surgical cavity. A biopsy was then performed of the left parotid mass, confirming metastatic SCC. The three month PET-CT following SRS to the brain was positive only for the parotid lesion. The patient did not want further surgery and elected for stereotactic ablative body radiotherapy. She received 35 Gy with 7 Gy per fraction. Figure 1 shows this plan in detail. Since that time, she has had no clinical or radiologic evidence of disease recurrence with multiple CT and PET CT’s performed. She is now almost 72 months removed from her initial diagnosis of metastatic HNSCC that was initially treated with palliative intent, with multiple subsequent therapies as outlined. Her last treatment with radiation to the parotid gland was over 42 months ago. The patient is alive, doing well, with no new or recurrent evidence of disease.

## Discussion

This case has many interesting features.  The patient was initially to be treated palliatively for metastatic HNSCC with chemotherapy and achieved a complete response. Upon relapse, there was no evidence of metastatic disease; thus, she was able to undergo definitive chemo-radiation for loco-regional disease with a complete response. Additionally, the location of the oligometastases in our patient was in the axilla, parotid gland, and intracranial cavity. It is well documented that the majority of metastases from HNSCC are found in the lung, which accounts for two-thirds of cases, with bone the next most common, followed by liver, while skin, mediastinum, and bone marrow are other less common sites [4-5]. In the past, this may not have been much of an issue, as the only options may have been palliative chemotherapy based on the location. However, local ablative therapy is being widely applied to various oligometastatic lesions, with most research pertaining to hepatic and pulmonary oligometastases [7, 9]. 

The increasing use of local ablative therapy from advancements in surgery and radiotherapy for oligometastatic disease raises the question on how best to manage such patients, as in our case, with the potential for curative intent being on the table for a stage of the disease once considered incurable and with low survival without treatment. Currently, patients are identified for possible local ablative curative-intent therapy based on risk factors, such as the number of metastases, interval to metastatic presentation, histology, and efficacy of systemic therapy [8]. One of the biggest challenges is to identify those patients who will really benefit from locally aggressive therapy.

As mentioned, the vast majority of evidence for the use of local ablative therapy is in lung and liver oligometastases, for which surgical resection has been the standard of care for many years and has been shown to prolong survival and decrease morbidity with a potential cure in selected individuals [7]. This local ablative strategy with surgery has led to the idea that stereotactic ablative body radiotherapy (SABR) may be as effective, with studies suggesting comparable local control rates and survival compared to surgery, with radiation patients typically being less fit [9]. 

We were unable to find any published literature on the usage of SABR for parotid oligometastases from HNSCC or from any primary site of disease for that matter. The standard treatment involves surgical resection with or without the addition of external beam radiation and the addition of chemotherapy in select individuals [6]. Our patient did not want a surgical procedure and opted for SABR alone to her localized oligometastatic lesion for which she had a complete response. She is currently 42 months removed from treatment with no evidence of disease at this site or any other location in her body.  

## Conclusions

To our knowledge, this is one of the first reported cases in the literature involving the use of SABR alone for oligometastatic disease to the parotid gland from any primary site, with a sustained complete response to therapy. The theoretical advantages of SABR over surgery could include no need for general anesthesia, lower morbidity, good immediate tolerance, and ambulatory treatment, along with the fact that certain patients may not be surgical candidates due to comorbidities. Furthermore, the use of SABR could limit the need for more aggressive systemic therapy, although current and future study results to further help risk stratify those patients most likely to benefit from SABR for long-term survival are warranted.
